# Use Your Judgment

**Published:** 1898-01

**Authors:** J. Y. Crawford


ARTICLE II.

### USE YOUR JUDGMENT.

BY DR. J. Y. CRAWFORD.

First deliberately question your judgment as to what is best to be done in each particular case. Do not drift into routine work, but study up each case as it presents.

Before you do anything (except for the immediate relief of pain) study the case well, to see which is best adapted of the various methods possible. In the same case one man would say, "Certainly, save those teeth," but another might say, "Remove them all." The great danger lies in specialism—all of one kind. Too many good dentists are inclined to avoid mechanical dentistry. We do not care to come in competition with the cheap, no account mechanical dentist of the Cheap John class, and in that very way we throw the work into the hands of that class. We must do more work along that line ourselves if we would cut him off. We must put a stop to that class of men who work only for the dollars, and who pull out teeth indiscriminately because they do not know how to do any thing else with them.

If dental surgery does nothing else than indoctrinate the medical world with a knowledge of the danger lying in the indiscriminate destruction of the human teeth it will have accomplished a great work. I advocate the modern system of crown- and bridge-work. Since its introduction it has doubled the work of high-class dentistry. We need a better class of mechanical dentists. It takes an artist to make a good set of artificial teeth. I want to commend the recent developments in swaging metal plates over a plaster die. It can accomplish results that are perfectly marvelous. Nothing can approximate a lower plate of aluminum, swaged over a plaster die, with rubber attachments. If the teeth are imperfectly occluded the lower plate moves and slips about, and hits the mucous membrane here and there, till the tissue becomes hypertrophied, and finally there remains only a soft bed for the plate to rest on. But there is less absorption under an aluminum plate swaged between two metal surfaces and driven home on a plaster die. It fits so beautifully that you can bring out the rugæ in an upper plate. You have all seen those soft, mushy jaws, where all the anterior part is like a second tongue. But you will have no more of that if you will adopt the swaged aluminum. I earnestly recommend the process of swaging metal plates over the plaster die, and assure you you need not break the model.—*Cosmos.*

---

ARTICLE III.

